# Editorial: Types of methods research papers in the journal *Campbell Systematic Reviews*

**DOI:** 10.1002/cl2.1172

**Published:** 2021-06-23

**Authors:** Ariel M. Aloe, Ruth Garside

**Affiliations:** ^1^ University of Iowa Iowa City USA; ^2^ University of Exeter Truro UK

Since its beginning in the early 2000s, the Campbell Collaboration has acknowledged the importance that methodology plays in producing systematic reviews. Indeed, the Methods Coordinating Group (CG) was one of the original groups in Campbell, alongside crime and justice, education, and social welfare. During the past two decades, numerous statisticians and methodologists have contributed to early forms of methods guidance and methods recommendations (it would be impossible to name them all and it would be unfair to just mention some). In recent years, with the expansion of the Campbell CGs, the type of questions asked and evidence used among CGs has diversified. These changes produce new challenges, and with new challenges new opportunities arise as well.

In more recent years, with the intention to better serve the rest of the CGs, the Methods CG, with the support of the board, the Editor in Chief, and the CEO, started to produce discussion papers, methods guidance, and methods policy. These early efforts are collected as Methods Research Papers in the *Campbell Systematic Reviews* journal.

Methods articles are open access, peer‐reviewed multidisciplinary manuscripts. The primary goal of the articles published within the *Campbell Systematic Reviews* journal is to provide methodological support for the systematic reviews and evidence and gap maps that it also publishes.

As such, we welcome different categories of manuscripts (described more fully below):

- Method: Innovative Methods Papers
- Research Methods Guide
- Method: Translations
- Research Methodology: Discussion Paper—Methodology
- Systematic Review: Systematic Reviews of Methods, and
- Guidance Paper
- Policy Paper

Methods manuscripts published in the *Campbell Systematic Reviews* journal are anchored in addressing practical problems of designing, conducting, reporting, and implementing systematic reviews and their results. We welcome manuscripts that use different forms of inquiry and traditions. Because we want to provide transparent and accessible methodological information to researchers, we encourage any data and source code used in the submission to be submitted with the manuscript prior to peer review. In cases in which quantitative manuscripts provide findings from a simulation study, the source code of the simulation should be provided. Although not required, we encourage all submissions that use software for analyses (quantitative or qualitative) to rely on an open‐source software. Finally, the Methods CG will disseminate guidance and policy papers as Guidance and policy papers.

## METHOD: INNOVATIVE METHODS PAPER

We welcome Innovative Methods Papers that introduce a novel approach to any of the stages of a systematic review, compare the use of different known methods, or demonstrate the accuracy or inaccuracy of a known method. Innovative Methods must be relevant to at least one of the stages of *Campbell Systematic Reviews*, or evidence and gap maps. These manuscripts must provide some examples (when feasible with real data), and the data and source code utilized in the example must be submitted at the time for the submission. If a simulation study is conducted, the source code of the simulation study must be included with the submission. For an example of an Innovative Methods Paper see Polanin and Nuijten (2018).

## RESEARCH METHODS GUIDE

There are papers that demonstrate, illustrate, and teach other researchers undertaking systematic review how to use specific methods. Whether focusing on a specific technique or software, these papers must be written in accessible language in order to reach a broader audience that may be interested in learning the practical use of the technique or software implemented. Because the goal of the tutorial is to aid other researchers in learning something that they have not yet mastered, particular attention should be given to the demonstration component of the manuscript. Papers must clearly articulate the objectives of the guide. Although not required, authors of Research Methods Guides are encouraged to create an appendix with a set of Q&A to help the reader test their understanding of the content. For an example of a Research Methods Guide see Papakonstantinou et al. (2020).

## METHOD: TRANSLATION

These are short (two to three pages) papers aimed at stakeholders and consumers of systematic reviews. These papers aim to communicate, in plain language, the reason that a particular method is used and why it matters for stakeholders and consumers. Translation Papers answer questions such as: What should a stakeholder know about a particular method? Why should stakeholders care that systematic reviews use a specific method? For example, what should a stakeholder know about heterogeneity? Why should stakeholders care that systematic reviews account for dependence?

## RESEARCH METHODOLOGY: DISCUSSION PAPER—METHODOLOGY

These are relatively short papers that discuss a specific method‐related topic. Discussion papers are not intended to be an exhaustive review of a topic or introduce a new method. This type of manuscript intends to highlight methods, such as specific advantages of a method or tool (e.g., software) and to explain the possible implications of the implementation of the discuss methods or tools. This type of paper is similar to commentary papers in other journals. For an example of a Research Methodology: Discussion Paper, see Haddaway et al. (2020).

## SYSTEMATIC REVIEWS OF METHODS

These are systematic reviews of methods. Systematic reviews of methods answer questions related, but not limited to: How is a particular method being used? What is reported when a particular method is being used? What is the overall bias or precision of a method while synthesizing multiple simulation studies? For examples of Systematic Reviews of Methods see Villar and Waddington (2019) and Wang et al. (2021).

## GUIDANCE PAPERS

The Methods CG encourages the constituents of the Campbell Collaboration to write guidance papers. Guidance Papers are recommendations in how to approach a particular issue. Although it may be considered good practice to follow a specific guidance, it is not mandated or expected that all Campbell reviews comply with these. Guidance papers are sent for consultation with all CGs as part of the peer review process. The guidance on information retrieval (Kugley et al., 2017) and on evidence and gap maps (White et al., 2020) are examples of guidance.

## POLICY PAPERS

Policies are, typically, previous guidance that has been elevated to policy. All Campbell reviews are expected to comply with policies. As part of the peer review process, Policy Papers are sent for consultation to all CGs. In addition, policies need to be approved by a technical panel. The Methodological Expectations for Campbell Collaboration Intervention Reviews (MECCIR) represent the Campbell policy on expectations (MECCIR Conduct Standards).

The inclusion of methods articles in the *Campbell Systematic Reviews* journal is another step that the Campbell Collaboration is taking to support the publication of high‐quality systematic reviews and evidence and gap maps. For questions related to the methods articles, please contact us at methods@campbellcollaboration.org.
